# Surgical intervention for paediatric liver injuries is almost history - a 12-year cohort from a major Scandinavian trauma centre

**DOI:** 10.1186/s13049-016-0329-x

**Published:** 2016-11-29

**Authors:** Tomohide Koyama, Jorunn Skattum, Peder Engelsen, Torsten Eken, Christine Gaarder, Pål Aksel Naess

**Affiliations:** 1 Department of Traumatology, Oslo University Hospital Ullevaal, PO Box 4950, Nydalen, N-0424 Oslo, Norway; 2 School of Medicine, University of Oslo, Oslo, Norway; 3 Department of Anesthesiology, Oslo University Hospital Ullevaal, Oslo, Norway; 4 University of Oslo, Oslo, Norway

**Keywords:** Children, Hepatic trauma, Nonoperative management, Angiographic embolization, Traumatic bile leak

## Abstract

**Background:**

Although nonoperative management (NOM) has become standard care, optimal treatment of liver injuries in children is still challenging since many of these patients have multiple injuries. Moreover, the role of angiography remains poorly defined, and a high index of suspicion of complications is warranted. This study reviews treatment and outcomes in children with liver injuries at a major Scandinavian trauma centre over a 12-year period.

**Methods:**

Patients <17 years old with liver injury admitted to Oslo University Hospital Ullevaal during the period 2002-2013 were retrospectively reviewed. Data were compiled from the institutional trauma registry and medical records.

**Results:**

A total of 66 children were included. The majority was severely injured as reflected by a median injury severity score of 20.5 (mean 22.2). NOM was attempted in 60 (90.9%) patients and was successful in 57, resulting in a NOM success rate of 95.0% [95% CI 89.3 to 100]. Only one of the three NOM failures was liver related, occurred in the early part of the study period, and consisted in operative placement of drains for bile leak. Two (3.0%) patients underwent angiographic embolization (AE). Complications occurred in 18 (27.3% [95 % CI 16.2 to 38.3]) patients. Only 2 (3.0%) patients had liver related complications, in both cases bile leak. Six (9.1%) patients underwent therapeutic laparotomy for non-liver related injuries. Two (3.0%) patients died secondary to traumatic brain injury.

**Discussion:**

This single institution paediatric liver injury cohort confirms high attempted NOM and NOM success rates even in patients with high grade injuries and multiple accompanying injuries. AE can be a useful NOM adjunct in the treatment of paediatric liver injuries, but is seldom indicated. Moreover, bile leak is the most common liver-related complication and the need for liver-related surgery is very infrequent.

**Conclusion:**

NOM is the treatment of choice in almost all liver injuries in children, with operative management and interventional radiology very infrequently indicated.

## Background

Nonoperative management (NOM) has become standard care in children with isolated blunt liver injuries based on experiences from institutions in North America [1, 2]. However, the role of some interventions, such as angiographic embolization (AE), is still uncertain [3]. Furthermore, the optimal treatment of bile leaks is still a matter of uncertainty. Another problem is posed by patients with other injuries that might challenge NOM of the liver injury. Yet, there is a paucity of reports from Europe addressing contemporary treatment of paediatric liver injuries.

The aim of the present study was to review the treatment and outcomes in children with liver injuries at a major Scandinavian trauma centre over a recent 12-year period to clarify whether our treatment practice and results are in accordance with international standards.

## Methods

All patients <17 years old with liver injury admitted at Oslo University Hospital Ullevaal (OUHU) from January 1st 2002 to December 31st 2013 were retrospectively identified from the institutional trauma registry. The trauma registry includes all trauma patients admitted through trauma team activation (irrespective of ISS), or with penetrating injuries proximal to elbow or knee, or with ISS ≥ 9 admitted to OUHU directly or via a local hospital within 24 h after injury. Transfers more than 24 h after injury are included only if the trauma team is activated. The study was approved by the Institutional Data Protection Officer.

Patients categorised as dead on arrival or who died within 30 min of arrival were excluded from further analysis, as were patients transferred from other hospitals later than 24 h after injury.

Demographic data, mechanism of injury (MOI), anatomic injury by body region (classified according to the Abbreviated Injury Scale (AIS) 98), Injury Severity Score (ISS), haemodynamic and physiological parameters on admission, admission laboratory values, diagnostic examinations, management strategies, and outcomes were compiled from the trauma registry and medical records of the patients.

As part of the study the computed tomographic (CT) scans were reviewed for liver injury severity and scored according to the Organ Injury Scale (OIS, 1994 revision) described by the American Association for the Surgery of Trauma [4].

Positive abdominal findings included: abdominal pain, signs of abdominal wall injury, peritonism, haematuria, melena or haematemesis.

Outcome data included NOM, failure of NOM (NOM-F), operative management (OM), AE as part of NOM, transfusions within the first 24 h, complications, intensive care unit (ICU) and in-hospital length of stay (LOS), and mortality.

NOM-F was defined as laparotomy performed after a period of attempted NOM. Angiography-related complications were defined as puncture site haematoma or pseudoaneurysm, intimal tears, unintentional coil migration, gallbladder necrosis, and allergic contrast reaction. Liver-related complications were defined as delayed liver haemorrhage, hepatic abscess, hepatic necrosis, bile leak, biloma, haemobilia, bilhaemia, and biliary fistulae.

To clarify whether NOM is achievable even in patients with severe liver injuries the subgroups of patients with high grade injuries (OIS grade IV-V) and patients with OIS grade I-III injuries were compared.

Categorical variables were presented as observed frequency with percentage, with addition of 95% confidence intervals (CI) for the overall NOM success and complication rates, and compared by Chi square or Fisher exact tests as deemed appropriate. Continuous variables were presented as median with inter quartile range (IQR), and subjected to the Mann-Whitney *U* test. All *p* values reported are two-sided, and *p* values <0.05 were considered to indicate statistical significance. Statistical analysis was performed using the IBM SPSS Statistics version 22 (International Business Machines Corp., New York, USA).

## Results

A total of 72 paediatric patients with liver injuries were admitted during the study period. Six patients were excluded from further analysis based on the aforementioned criteria.

The MOI was blunt in 64 (97.0%) of the 66 included cases secondary to (only categories with more than one patient included): falls (22.7%, *n* = 15), motor vehicle crashes (MVC) (19.7%, *n* = 13), bicycle accidents (15.2%, *n* = 10), ski or snowboard activities (12.1%, *n* = 8), pedestrian accidents (10.6%, *n* = 7), and all-terrain vehicle (ATV) crashes (4.5%, *n* = 3).

Of the 66 included patients, nine (13.6%) had OIS grade I injury, 23 (34.8%) had grade II injury, 17 (25.8%) had grade III injury, 16 (24.2%) had grade IV injury, and one (1.5%) had a grade V injury.

Patient characteristics and outcome data for the total cohort and stratified by liver injury severity are presented in Table 1. Most patients were severely injured as reflected by a median ISS of 20.5 (10; 33), time spent in ICU 2 (1; 5) days, and overall LOS 7 (5; 11.3) days. A total of 58 (87.9%) patients had combined injuries: 15 patients had head injuries, 19 had facial injuries, five had spinal injuries, 35 had chest injuries, 27 had other abdominal injuries, and 21 had injuries to the pelvis or extremities in addition to the liver injury.

**Table 1:** Patient demographics and outcomes for the total cohort and stratified by liver injury severity

	All	OIS I-III	OIS IV-V	*p* value
	(*n* = 66)	(*n* = 49)	(*n* = 17)	
Age	10.5 (6.8; 14.1)	10.9 (6.8; 14.2)	10.0 (7.1; 13.1)	0.67
Male gender	43 (65.2)	32 (65.3)	11 (64.7)	0.96
Blunt injury	64 (97.0)	47 (95.9)	17 (100)	1.00
ISS	20.5 (10.0; 33.0)	17.0 (9.0; 28.0)	25.0 (17.0; 34.0)	0.02
GCS	15.0 (14.0; 15.0)	15.0 (14.0; 15.0)	15.0 (15.0; 15.0)	0.16
Base excess^a^	−2.1 (−4.9; −1.1)	−2.4 (−4.7; −0.9)	−2.0 (−5.3; −1.4)	0.96
Lactate^b^	2.2 (1.3; 2.9)	2.3 (1.3; 2.9)	1.8 (1.4; 2.9)	0.55
Aspartate transaminase^c^	302.5 (161.3; 521.0)	261.0 (145.0; 357.0)	618.0 (416.5; 969.0)	<0.01
Alanine transaminase^d^	220.0 (120.0; 390.0)	189.5 (110.8; 266.5)	461.0 (355.0; 723.0)	<0.01
Positive abdominal findings	45 (68.2)	29 (59.2)	16 (94.1)	0.01
Attempted NOM	60 (90.9)	45 (91.8)	15 (88.2)	0.64
Angiographic embolization	2 (3.0)	0 (0.0)	2 (11.8)	0.06
Patients transfused	14 (21.2)	10 (20.4)	4 (23.5)	0.74
ICU LOS	2.0 (1.0; 5.0)	2.0 (1.0; 5.0)	2.0 (1.5; 7.0)	0.84
In-hospital LOS	7.0 (5.0; 11.3)	6.0 (5.0; 11.5)	7.0 (6.0; 13.0)	0.18
Mortality	2 (3.0)	1 (2.0)	1 (5.9)	0.45

Values are given as median (first and third quartiles) or n (%) as appropriate

*OIS* organ injury scale, *ISS* injury severity score, *GCS* Glasgow coma scale, *NOM* nonoperative management, *ICU* intensive care unit, *LOS* length of stay

^a^Data from 50 patients

^b^Data from 36 patients

^c^Data from 62 patients

^d^Data from 63 patients

As expected, children with grade IV-V injuries had higher median ISS, aspartate transaminase (AST) levels, and alanine transaminase (ALT) levels than patients with grade I-III injuries.

NOM was attempted in 60 (90.9%) patients and was completed in 57 resulting in a NOM success rate of 95.0% [95% CI 89.3 to 100]. All NOM-F occurred in the first two years of the study period, and only one was liver related. Two NOM-F were not liver related. In both cases, laparotomy was performed for associated abdominal injuries, one resulting in splenectomy and the other in distal pancreatectomy.

Only two patients underwent angiography due to their liver injuries. Both had been subjected to a handle bar injury and were transferred from a local hospital after CT scan showed high grade liver injury. They both showed clinical signs of bleeding within the first 24 h after admission and underwent angiography followed by successful selective AE of branches of the right hepatic artery.

Six (9.1%) children with liver injury underwent OM. Four patients had non-liver related therapeutic laparotomy performed based on clinical and/or radiological findings: three had repair of hollow viscus injuries (HVI) and one had a repair of a diaphragmatic rupture. Two children underwent planned second look surgery with abdominal closure after damage control surgery in local hospitals prior to transfer.

Complications occurred in 18 (27.3% [95% CI 16.2 to 38.3]) patients. Only two patients had liver related complications, in both cases bile leak. The first patient with bile leak was treated with percutaneous drainage on day 11. The leak gradually decreased and the drain could be successfully removed after 11 days. The second patient was one of the NOM-F previously mentioned. He was diagnosed with a bile leak on post injury day 6 that was drained percutaneously followed by operative placement of perihepatic drains which were subsequently removed with no further complications. There was no need for endoscopic interventions.

Two (3.0%) patients died secondary to severe traumatic brain injury (TBI).

## Discussion

This single institution paediatric liver injury cohort demonstrates high attempted NOM and NOM success rates even in patients with high grade injuries and multiple accompanying injuries. AE can be a useful NOM adjunct in the treatment of paediatric liver injuries, but is seldom indicated. Moreover, bile leak is the most common liver-related complication and the need for liver-related surgery and endoscopic interventions is very infrequent.

Most of the patients (87.9%) had multiple injuries as reflected in the high median ISS of 20.5, which is slightly higher than other studies [5–7]. This is also reflected in days spent in the ICU and overall LOS. Although the overall mortality of 3.0% in our cohort was lower than reported in the aforementioned studies, this probably reflects different exclusion criteria and does not allow any speculations about differences in the quality of care. The main cause of death was TBI as in most paediatric trauma cohorts [5–11].

Although many studies report MVCs to be the most common MOI [6, 11–14], fall was the leading MOI in our population. Ski and snowboard activities caused 12.1% of the injuries, probably reflecting the popularity of winter sports in Norway. ATV-related injuries in children have received much attention [15–17], in our cohort three (4.5%) patients were involved in ATV crashes.

Elevated serum levels of AST and ALT seem to be a reliable indicator of liver injury in children within the first hours after trauma [18–20]. In accordance with this, both AST and ALT levels were markedly increased and correlated to OIS-grading (Table 1). Children with grade IV-V injuries had significantly higher levels of AST and ALT on admission compared to children with grade I-III injuries.

NOM of hepatic injuries is the standard of care in haemodynamically normal(ised) paediatric patients [1, 2]. Such patients tolerate diagnostic work-up with CT scan. Unless other injuries that mandate laparotomy are verified (e.g., free intraabdominal air indicating hollow viscus perforation), NOM of the liver injury is attempted. In spite of a slightly higher ISS in this study, the success rate of NOM was high at 95.0%, which is in accordance with other published studies [6, 10, 11, 21].

Reimaging of the liver injury is only performed on clinical suspicion of complications, e.g., suspected bleeding or bile leak. In patients with isolated liver injuries the American Pediatric Surgical Association guidelines are followed [1]. According to these guidelines routine ICU admission for 24 h is reserved for patients with high grade (≥ grade IV) injuries only and proposed hospital stay is one day longer than OIS injury grade. We do not practice mandatory bed rest. In the ward recording of blood pressure and pulse rate is done every fourth hour the first 24 h and then three times/24 h until discharge. Activity restriction after discharge is two weeks longer than OIS injury grade and return to full-contact, competitive sports should be individualized [1].

Despite the high NOM success rates, there is still concern about of missing HVIs when NOM is standard care. However, the reported overall rates of HVI in children with blunt abdominal trauma are as low as 0.8–3.2% [22–24]. In the present study, the three (4.5%) patients with suspected HVIs based on clinical and radiological findings all underwent primary OM and the diagnosis was confirmed perioperatively.

The existing literature on AE as an adjunct treatment to paediatric liver injuries is limited to recent case series [25–27]. In our cohort, only two (3.0%) patients underwent AE, but the procedure was therapeutic in both cases and further transfusions and more invasive surgical procedures were thus avoided. In our opinion AE as an adjunct to NOM should be considered only in patients with a CT verified liver injury with extravasation, and who develop clinical signs of bleeding.

Liver related complications are rare (0.0–8.0%) and the main cause is bile leak, as reported by several authors [5, 11, 28, 29]. The development of new symptoms, including increasing abdominal pain, distention, nausea, and vomiting will prompt suspicion in the majority of cases [29]. In our cohort, two (3.0%) patients underwent drainage procedures due to bile leak. In the first year of the study period one patient underwent operative placement of perihepatic drains due to ongoing bile leak after removal of percutaneous drains. In the later part of the period the second patient with bile leak underwent curative percutaneous drainage. Kulaylat et al. presented the largest single-institution series with bile leaks from blunt liver injury in children in 2014 [29]. Based on their successful experience with 11 patients they recommended percutaneous drainage combined with endoscopic retrograde cholangiopancreaticography (ERCP) with sphincterotomy and/or biliary stenting. Whether ERCP related procedures increase the success rate of percutaneous drainage cannot be established from that study. ERCP is an invasive procedure with associated complications, and our experience with adult and paediatric liver injuries is that bile leaks stop eventually spontaneously, without the need for ERCP.

This study has limitations in addition to its retrospective nature. It is a single-institution study describing a limited number of children with liver injuries over a 12-year period. However, the reported data clearly demonstrate high attempted NOM and NOM success rates in a cohort of severely injured patients.

## Conclusion

This single institution paediatric liver injury cohort confirms high attempted NOM and NOM success rates even in patients with high grade injuries and multiple accompanying injuries. AE can be a useful NOM adjunct in the treatment of paediatric liver injuries, but is seldom indicated and should be considered only in patients with a CT verified liver injury with extravasation, and who develop clinical signs of bleeding. Moreover, bile leak is the most common liver-related complication, should be treated with percutaneous drainage, and the need for liver-related surgery is very infrequent.

## Abbreviations

AE: Angiographic embolization
AIS: Abbreviated injury scale
ALT: Alanine transaminase
AST: Aspartate transaminase
ATV: All-terrain vehicle
CI: Confidence interval
CT: Computed tomography
HVI: Hollow viscus injury
ICU: Intensive care unit
ISS: Injury severity score
LOS: Length of stay
MOI: Mechanism of injury
MVC: Motor vehicle crash
NOM: Nonoperative management
NOM-F: Failure of nonoperative management
OIS: Organ injury scale
OM: Operative management
OUHU: Oslo University hospital Ullevaal
TBI: Traumatic brain injury
